# Identification of POLQ as a chromosomal instability-associated biomarker for hepatocellular carcinoma

**DOI:** 10.1016/j.gendis.2025.101553

**Published:** 2025-02-04

**Authors:** Ying Cai, Na Wang, Qiang Liu, Yidi Wang, Dina Liu, Jing Liu, Hengyu Zhou

**Affiliations:** a School of Nursing, Chongqing Medical University, Chongqing 400016, China; b Key Laboratory of Molecular Biology for Infectious Diseases (Ministry of Education), Chongqing Medical University, Chongqing 400016, China; c Key Laboratory of Molecular Biology for Infectious Diseases (Ministry of Education), Institute for Viral Hepatitis, Department of Infectious Diseases, The Second Affiliated Hospital, Chongqing Medical University, Chongqing 400010, China; d Department of Hepatobiliary Surgery, The First Affiliated Hospital of Chongqing Medical University, Chongqing 400016, China; e Department of Breast and Thyroid Surgery, The First Affiliated Hospital of Chongqing Medical University, Chongqing 400016, China; f Department of Pathogen Biology, College of Basic Medical Science, Chongqing Medical University, Chongqing 400016, China

Chromosomal instability (CIN) drives cancer development by causing the accumulation of significant gains, losses, and rearrangements of DNA, although the degree to which this impact relies on DNA damage is uncertain in terms of mechanisms and key molecules. Nevertheless, a structured system for evaluating various forms of CIN and their impact on hepatocellular carcinoma (HCC) is lacking. Here we evaluated CIN as a hallmark of HCC with LASSO risk prognostic model. Our framework figured out that DNA polymerase theta (POLQ) facilitated CIN in HCC and exhibited higher levels of expression in HCC compared with normal tissues. Moreover, the increased POLQ expression was closely linked to the poor prognosis of patients with HCC. This biomarker indicates a negative outlook, worsening the irregularities in spindle formation, chromosomes, and chromosome bridges, elevating the protein levels of key spindle assembly checkpoint components, and promoting the cell cycle and proliferation of HCC cells. Our results demonstrate an excellent diagnostic model in distinguishing CIN in HCC, and the POLQ promoted CIN and could guide the clinical diagnosis, prognosis, and therapy in HCC. The findings could offer fresh perspectives on tailored therapy for HCC and improve the prognosis of HCC patients.

HCC is one of the most common malignant tumors in the world, which is frequently diagnosed at a late stage with a poor prognosis. It is a heterogeneous group of tumors that vary in terms of risk factors and genetic and epigenetic alteration event.^1^ Nevertheless, the newly developed therapeutic strategies have not achieved universal success and HCC patients frequently exhibit therapeutic resistance. It is necessary to identify biomarkers reflecting the HCC progression for guidance of HCC diagnosis, prognosis prediction, and progression block. CIN is the most prevalent form of genomic instability, a well-established hallmark of cancer cells, and is detectable in precancerous lesions and associated with disease stage, metastasis, poor prognosis, and therapeutic resistance.^2^ The causes of CIN are also diverse and include mitotic errors, replication stress, homologous recombination deficiency, telomere crisis, and breakage fusion bridge cycles, among others. Studies indicate that CIN exists in cirrhotic liver and dysplastic nodules and more than 90% of HCC cases.^3^ Therefore, CIN-associated genes may be a promising focus for depicting the progression of HCC, and targeting CIN may be a promising therapy for HCC.

Firstly, using datasets (GSE54236, GSE62232, GSE112790, and GSE121248) obtained from the Gene Expression Omnibus (GEO) repository, we analyzed differentially expressed genes in HCC samples compared with normal samples. Subsequently, an enrichment analysis of 31 genes derived from the overlapping results indicated that CIN is an important characteristic of HCC (Fig. S1A; Fig. 1A). For further exploration of CIN in HCC, a risk score model predicting HCC patient survival based on Least Absolute Shrinkage and Selection Operator regression (LASSO) Cox regression was constructed based on these genes from TCGA datasets (Fig. 1B–D). Survival analysis showed the high-risk HCC group's overall survival, disease-specific survival, and progression-free interval were considerably poorer than those of the low-risk HCC group (Figs. S1B–D). It is suggested that the model risk score has a good differentiation degree for the prognosis of HCC. The risk scores obtained from the prognostic model were analyzed for their correlation with the clinical presentation of patients with HCC. The risk scores increased significantly with the progression of the clinical T-stage, pathological stage, pathological grading, tumor free (the patient was tumor-free up to the follow-up cut-off time), the levels of alpha-fetoprotein, fibrosis Ishak score, and the status of vascular invasion (Fig. S1E–L).

**Figure 1:**
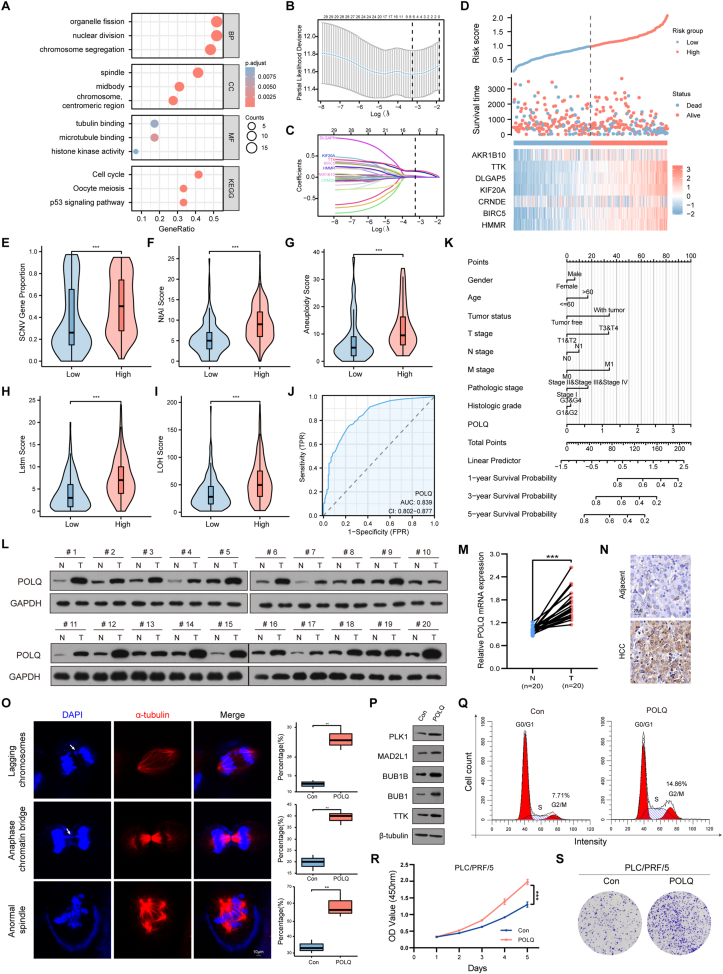
POLQ is associated with CIN, progression, and prognosis in HCC. **(A)** Gene ontology (GO) and Kyoto Encyclopedia of Genes and Genomes (KEGG) enrichment analysis for 31 differentially expressed genes. **(B**–**D)** LAASO coefficient screening diagram and LASSO variable trajectory diagram were narrowed down by the Lasso Cox regression algorithm, employing five-fold cross-validation. **(E**–**I)** Comparisons of CIN (SCNV, NtAI, LOH, LSTm, and aneuploidy scores) between the high and low POLQ expression groups (∗∗∗*p* < 0.001). SCNV, somatic copy number variation gene proportion; NtAI, the number of telomeric allelic imbalances; LOH, loss of heterozygosity; LSTm, large-scale state transitions. **(J)** The receiver operating characteristic curve shows the predictive value of POLQ in HCC. **(K)** The nomogram shows the POLQ-related HCC prediction model. **(L)**Western blot analysis for POLQ protein expression level in HCC and paired adjacent normal liver tissues. N, normal; T, tumor. **(M)** Quantitative reverse transcription PCR analysis for POLQ mRNA expression level in paired HCC and adjacent normal liver tissues (mean ± standard deviation, ∗∗∗*p* < 0.001, student's *t*-test). **(N)** Immunohistochemistry analysis for the expression level of POLQ in HCC and paired adjacent normal liver tissues (scale bars, 20 μm). **(O)** Immunofluorescence analysis of α-tubulin (red) revealed that overexpression of POLQ led to delayed chromosome separation and chromosome bridge. DAPI (blue) was used to stain the nuclei. Scale bars, 10 μm. **(P)** Western blot of the expression level of spindle assembly checkpoint-related proteins with POLQ overexpression. **(Q)** The distribution of different cell cycle phases was determined by flow cytometry. Cell cycle analysis revealed that the proportion of the G2/M phase significantly increased in POLQ overexpression cells relative to control cells. **(R, S)** CCK-8 assay and clone formation in POLQ overexpression cells. POLQ, DNA polymerase theta; CIN, chromosomal instability; HCC, hepatocellular carcinoma.

To further investigate how the prognostic model works, the correlation between DNA damage repair-related genes and risk scores of the HCC clinical prognostic model was analyzed. The analysis showed that POLQ was strongly associated with the highest correlation on risk scores among the entire family of DNA polymerases (spearman's correlation coefficient greater than 0.8, *p* < 0.001) (Fig. S2A). POLQ, a DNA repair enzyme known as DNA polymerase theta, assists in the pairing and elongation of 3′-ssDNA overhangs formed after resection of a double-stranded DNA break site using microhomology tracts.^4^ The POLQ exhibited substantial positive correlations with five genes (DLGAP5, KIF20A, TTK, BIRC5, and HMMR) from LASSO Cox regression, and the group with high POLQ expression had greater levels of somatic copy number variation (SCNV) gene proportion, the number of telomeric allelic imbalances (NtAI), loss of heterozygosity (LOH), large-scale state transitions (LSTm), and aneuploidy scores (Fig. S2B; Fig. 1E–I). Functional enrichment analysis indicated their enrichment in chromosome and nuclear chromosome segregation processes, in cellular components like chromosome, spindle, and centromeric region, and Kyoto Encyclopedia of Genes and Genomes (KEGG) pathway association with cell cycle and DNA replication (Fig. S2C). An upset plot mapped the correlation between POLQ and related genes such as BUB1B, BUB1, TTK, CDC6, MAD2L1, PLK1, CCNB1, and CDC20 (Fig. S2D, E). Gene set enrichment analysis (GSEA) of differentially expressed genes associated with POLQ revealed that the functional enrichment results mainly focused on the mitotic spindle checkpoint, DNA double-strand break repair, DNA repair, and gene sets related to the cell cycle (Figs. S2F–I).

Additionally, pan-cancer analysis was performed to confirm the importance of POLQ (Fig. S3A). The protein and RNA levels of POLQ in HCC tumors were significantly elevated than those of the adjacent normal liver tissues (Fig. 1L–N; Fig. S3B). Furthermore, there is a notable rise in POLQ expression as clinical T-stage, pathological stage, and histologic grade in HCC patients (Fig. S3C–E), and the overall survival, disease-specific survival, and progression-free interval in HCC patients were linked to the high POLQ expression level (Fig. S3F–H). The area under the receiver operating characteristic curve was 0.839, indicating a high degree of specificity and sensitivity (Fig. 1J). A prognostic nomogram was established based on clinical indicators and predicted the 1-, 3- and 5-year overall survival of liver cancer patients with a concordance index of more than 0.7 from calibration (Fig. 1K; Fig. S3I). The mutations in TP53, RB1, TSC2, and ROS1 promote increased POLQ expression levels, and the effect of TP53 mutations on POLQ in other cancers (Fig. S4A–L), suggesting that these mutated genes affect DNA damage repair and promote genomic instability.

Finally, the effects of POLQ on chromosome segregation, cell cycle, and proliferation were examined in human HCC cell lines. The results revealed that the spindle formation was abnormal in the middle stage of mitosis, and delayed chromosome and chromosome bridge appeared in the late stage by immunofluorescence, which are often considered important markers of CIN in POLQ overexpression HCC cells (Fig. 1O; Fig. S5A). Meanwhile, the protein levels of BUB1B, BUB1, TTK, MAD2L1, and PLK1, important components of spindle assembly checkpoint were also examined (Fig. 1P). In eukaryotic cells, spindle assembly checkpoint is an important monitoring mechanism to ensure the fidelity of mitotic chromosome separation. Such elevated gene expression overactivated anaphase-promoting complex/cyclosome activity, leading to prolonged mitosis and increasing the incidence of lagging chromosomes.^5^ This further suggested that the high expression level of POLQ might lead to CIN. The flow cytometry analysis showed that the increased expression of POLQ led to an elevated proportion of cells in the G2/M phase, while the opposite trend in cells knocked down POLQ expression (Fig. 1Q; Fig. S5B, C). The extension of the G2 phase may be closely associated with abnormal chromosome segregation. The CCK-8 and clone formation assay showed that whereas POLQ silencing suppressed proliferation, POLQ overexpression increased HCC cell proliferation (Fig. 1R; Fig. S5D, E).

In conclusion, we developed a prognostic model for HCC via risk score and our data showed that POLQ displayed as a biomarker connected to HCC progression and associated with CIN which enables guide clinical diagnosis, prognosis, and therapeutic. It is noteworthy that the precise role of POLQ in CIN within the context of HCC has not been previously elucidated, marking a significant contribution and innovative aspect of our study. The risk score was validated in multiple independent cohorts and could be regarded as an independent indicator of HCC prognosis. The diagnostic models showed excellent performance in distinguishing HCC from CIN. The expression levels of POLQ gradually increase during HCC progression. Elevated POLQ expression exacerbated CIN and enhanced the proliferation of HCC cells. In summary, these findings have a significant impact on clinical decision-making and offer hopeful new approaches for enhancing the prognosis of individuals with HCC.

## Ethics declaration

At Chongqing Medical University's Second Affiliated Hospital (Chongqing, China), liver tumor samples and adjacent non-tumor samples were taken from HCC patients undergoing hepatic resection. The hospital's committee (No. 2021-219) approved the procedure ethically following the Declaration of Helsinki. Every subject gave their informed consent.

## Funding

This study was supported by grants from the 10.13039/501100001809National Natural Science Foundation of China (No. 82403106, 82100217), 10.13039/100012546Chongqing Postdoctoral Science Foundation of China (No. cstc2021jcyj-bshX0118), 10.13039/501100005230Natural Science Foundation of Chongqing, China (No. cstc2021jcyj-msxmX0292, CSTB2024NSCQ-MSX0179), Scientific and Technological Research Program of Chongqing Municipal Education Commission (China) (No. KJQN202300451), Chongqing Medical University Future Medical Youth Innovation Team Support Program (China) (No. W0008), and Program for Nursing Collaborative Innovation, Chongqing Medical University, Chongqing, China (No. 20240203, 20240211).

## CRediT authorship contribution statement

**Ying Cai:** Writing – original draft, Visualization, Project administration, Investigation, Funding acquisition, Formal analysis. **Na Wang:** Resources, Funding acquisition. **Qiang Liu:** Funding acquisition, Data curation. **Yidi Wang:** Data curation. **Dina Liu:** Software, Investigation. **Jing Liu:** Writing – review & editing, Writing – original draft, Project administration, Investigation, Formal analysis. **Hengyu Zhou:** Writing – review & editing, Project administration, Funding acquisition.

## Conflict of interests

The authors declared no competing interests.

## Figures and Tables

**figs1:**
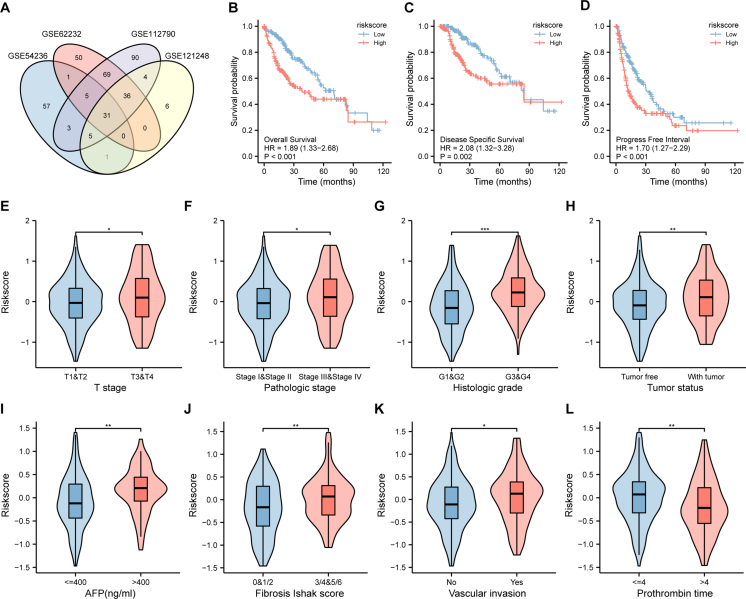


**figs2:**
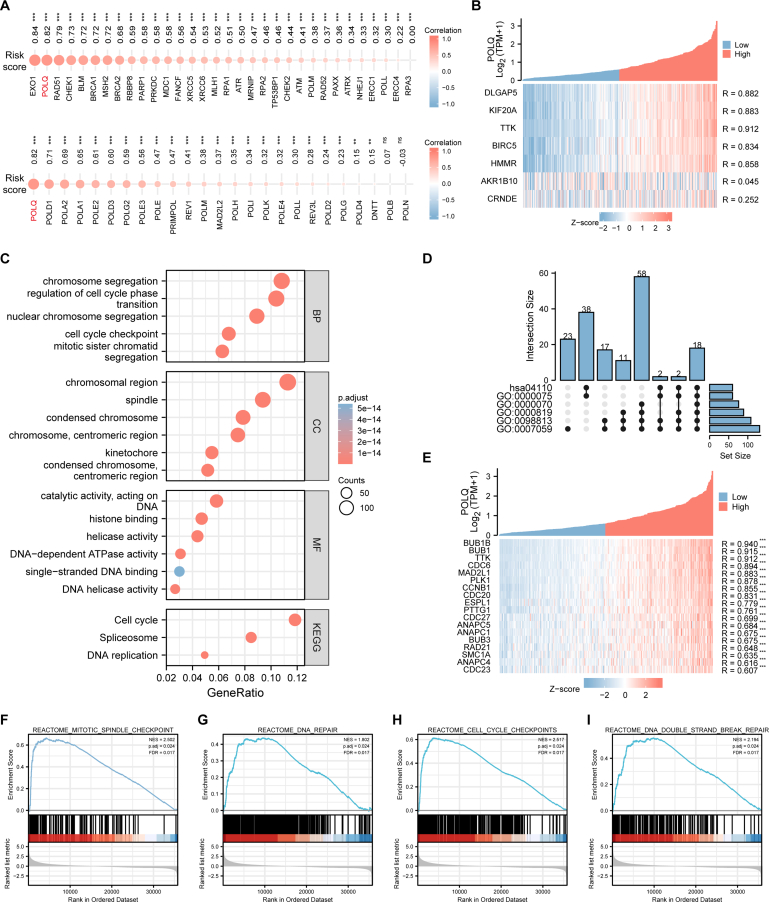


**figs3:**
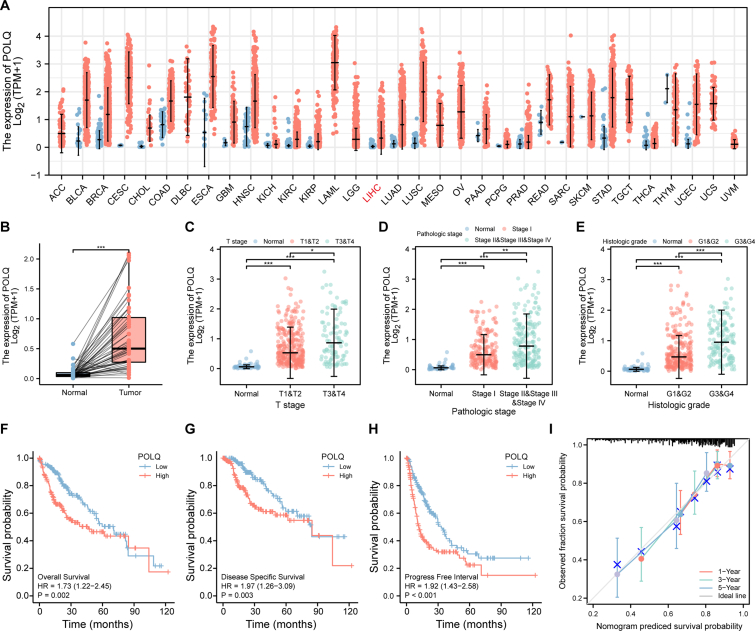


**figs4:**
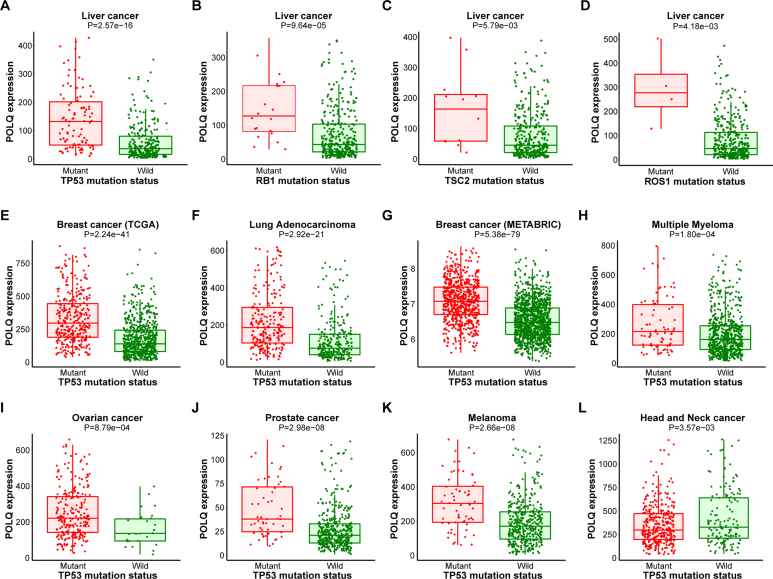


**figs5:**
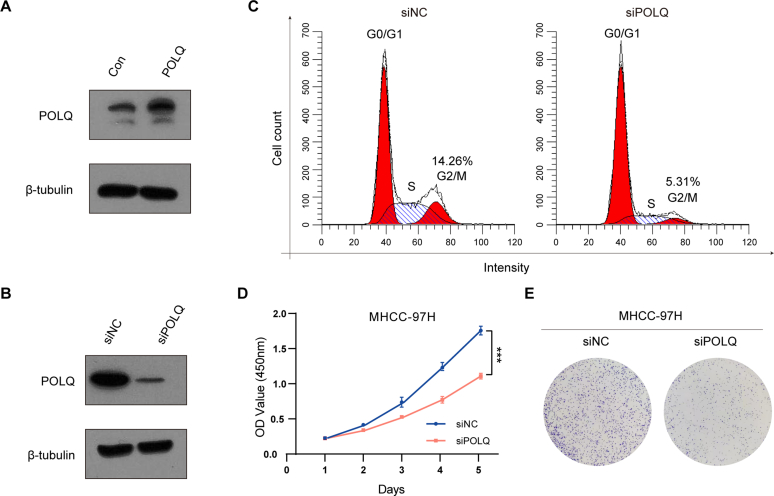


## Data Availability

The gene expression datasets GSE54236, GSE62232, GSE112790, and GSE121248 were obtained from the GEO database (https://www.ncbi.nlm.nih.gov/geo/). According to the TCGA (https://portal.gdc.cancer.gov/) liver hepatocellular carcinoma (LIHC) project, the differential RNA sequencing expression data in paired and unpaired samples were in level 3 HTSeq-FPKM forma. The data regarding chromosomal instability analysis were obtained from the cBioPortal (https://www.cbioportal.org/). The receiver operating characteristic curve analysis of liver hepatocellular carcinoma was derived from the TCGA_GTEx-LIHC dataset (https://xena.ucsc.edu/). The genetic mutations analysis was performed using muTarget tool in Kaplan–Meier Plotter (https://www.mutarget.com/).
