# Supplementary material for: Identification of POLQ as a chromosomal instability-associated biomarker for hepatocellular carcinoma
Source: Genes Dis. 2025 Feb 4;12(6):101553. doi: 10.1016/j.gendis.2025.101553 (PMC12304679; doi:10.1016/j.gendis.2025.101553)
Supplement: Multimedia component 1 [file mmc1.docx]

**Supplementary data**

**Material and methods**

**Data acquisition and analysis**

Using GEOquery package ^1^, the gene expression datasets GSE54236, GSE62232, GSE121248, and GSE112790 were withdrawed from the database of Gene Expression Omnibus (GEO) (<https://www.ncbi.nlm.nih.gov/geo/>). We used R software (Version: R x64) for data preprocessing ^2^. Eliminate probes that match with more than one molecule per probe. When multiple probes relate to a single molecule, only the one with the greatest value is kept. Differentially expressed genes (DEGs) were analyzed utilizing the “limma” package, which identified DEGs with logFC ≥ 1.5 and adjusted *p*-value (adj.*p*.val) <0.05.

According to the dataset of liver hepatocellular carcinoma (LIHC) project in TCGA (<https://portal.gdc.cancer.gov/>), the differential RNAseq expression in paired and unpaired samples were in level 3 HTSeq-FPKM forma ^3^. The FPKM (Fragments Per Kilobase per Million) format of RNAseq data was transformed to TPM (transcripts per million reads) format, and then log2 converted, while filtering out adjacent normal liver samples, eliminating duplicates, and preserving clinical information. The data used for the final analyses was formatted in TPM. Utilizing the clusterProfiler package (Version 3.14.3) and the org.Hs.eg.db package (Version 3.10.0) to examine Gene Ontology (GO) and Kyoto Encyclopedia of Genes and Genes (KEGG) enrichment, an analysis of DEGs was carried out with the purpose of conducting a more thorough functional study of hepatic expression profiles. The survival package (Version 3.2-10) and glmnet R package (Version 4.1-2) were utilized to perform LASSO logistic regression, identifying the optimal number of genes through five-fold cross-validation based on the minimal lambda value. The survival package and survminer package (Version: 0.4.9) were used to construct the Kaplan-Meier curve. The R package "pROC", "rms", "survival", "UpSetR", and "ggplot2" were adapted to create nomogram, receiver operating characteristic (ROC) curve, calibration, UpSet, and venn plots. c2.cp.v7.2.symbols.gmt (Curated), a curated reference gene collection from MSigDB Collections (https://www.gsea-msigdb.org/gsea/msigdb/collections.jsp) ^4, 5^, was used to conduct Gene Set Enrichment Analysis (GSEA) utilizing the DEGs of POLQ. If the adj.*p*.val and false discovery rate are less than 0.05 and 0.25, respectively, then enrichment is generally regarded as significant.

**Cell lines**

PLC/PRF/5and MHCC-97H were obtained from the Key Laboratory of Molecular Biology on Infections Disease, Ministry of Education (Chongqing, China). Cells were grown in Dulbecco′s Modified Eagle′s Medium (DMEM) (Sigma-Aldrich, USA) with 100 mg/ml of streptomycin, 100 U/ml of penicillin, and10% fetal bovine serum. Cells were cultured in a humidified incubator (Thermo Fisher Scientific, USA) with a consistent 5% CO_2_ at a temperature of 37 °C.

CRISPR/dCas9 SAM system and cell transfection

CRISPR/dCas9 Synergistic Activation Mediator (SAM) system was employed to increase the expression of POLQ in PLC/PRF/5 cells ^6^. Lentiviral vectors encoding dCas9-VP64 (LV-dCas9-VP64) and POLQ-targeting sgRNA (LV-POLQ-sgRNA) were purchased from Genechem (Shanghai, China). Following transfected with LV-dCas9-VP64, cells were selected with puromycin (Abcam, ab141453, UK) and subsequently infected with LV-POLQ-sgRNA. G418 (Sangon Biotech, B540723, China) was used for the selection of cells with targeted gene activation. The POLQ-sgRNA sequence was 5’-CGAAAGAGACAAGCTACTAT-3’.

**Transient transfection of small interfering RNA**

The POLQ mRNA is the target of small interfering RNA (siRNAs) that were purchased from Ribobio (Guangzhou, China). Following the instructions, MHCC-97H cells were underwent siRNAs with transfection reagent Lipofectamine 3000 (Invitrogen, L3000015, USA). After transfection, the original medium was swapped out for new medium, and MHCC-97H cells were cultivated for 48 hours before being studied in more detail. **Supplementary Table 1** lists the POLQ gene's target sequences.

**Immunoblotting**

Liver samples from HCC patients undergoing hepatic resection were taken from the tumor and adjacent to non-tumor livers at Chongqing Medical University's Second Affiliated Hospital (Chongqing, China). The hospital's committee approved the procedure ethically adhering to the principles of the Declaration of Helsinki (No. 2021-219). Every subject gave their informed consent. Using RIPA-buffer with complete protease inhibitors (Roche, USA), protein extracts were made, and measured with the BCA kit (Cwbio, CW0014S, Jiangshu, China). 30 μg of cell lysate was electrophoretically separated on SDS-PAGE gel and transferred to PVDF membranes (Merck Millipore, USA). Membranes were probed with primary antibodies overnight at 4 °C after being blocked for 1 hr at room temperature with 5% non-fat milk. Then the membranes were incubated with the secondary antibodies with horseradish peroxidase (**Supplementary Table 2**) at room temperature after washing, and finally were exposed to an X-ray film in a dark room.

**Cell Viability Assay**

Briefly, 1,500 cells were seeded in each well of 96-well plates and preincubated overnight to adhere under physiological conditions of 5% CO_2_ and 37℃. The cells were then treated with transfection of plasmids or siRNA. Each well was added 10 µl of CCK-8 (Selleck, B34302, USA) and incubated at 37℃ for 2 hours on days 0, 1, 2, 3, 4, and 5, then measured at 450 nm absorbance using BioTek Synergy HTX Multimode Reader (Agilent, USA). Each experiment was performed in triplicates, and the results were the mean of at least three independent measurements.

**Colonogenic assay**

Following transfection, PLC/PRF/5 and MHCC-97H cells were planted in 6-well plate and grown for 14 days. 4% polyformaldehyde (PFA) was used to fixed the cells, 0.4% crystal violet was used to dye. Colonies were counted with greater than 60 μm in size and the colony numbers were standardized by establishing the colony count of the control vector as 100%. The transfection experiment above was conducted thrice, with triplicate transfections for each vector on every occasion.

**Immunohistochemistry**

HCC specimens embedded in paraffin were deparaffinized and retrieved antigen were with 10 mM citrate buffer (pH 6.8) in a microwave oven for 2.5 min. Then slides were blocked with 3% H_2_O_2_ solution. Following the blocking step, diluted primary antibodies were applied to the slides, then left to incubate in a humidified chamber at 4 °C overnight. Subsequently, incubated a biotinylated secondary antibody at room temperature for 1 hr. Freshly prepared DAB substrate solution (ZSGB-BIO, Beijing, China) was utilized to visualize the antibody staining color. Nuclei were localized by hematoxylin staining for 1-2 min before dehydrating, mounting and capture.

**Immunofluorescence staining for spindle and chromosome bridge**

The cells were cultured in 12-well slide medium and added to nocodazole (100ng/ml) (Selleck, S2775, USA) for synchronization. After 14 hours of culture, nocodazole was removed and released into G2/M phase. Then cells were fixed in 4% polyformaldehyde, permeabilized and blocked. Following an overnight incubation at 4°C with anti-α-tubulin antibody (1:250) (Abcam, ab7291, UK), the samples were incubated for 1 hr at room temperature with TRITC-conjugated goat anti-Mouse IgG (1:1000) (ZSGB-BIO, ZF-0313, Beijing, China), and sealed with an anti-fluorescence attenuation tablet (DAPI) (Solarbio, S2110, Beijing, China). Leica TCS SP8 X Confocal Microscope (Leica, Germany) fluorescence imaging was used for signal identification.

**Cell cycle synchronization and flow cytometry (FCM) analysis**

The cells were cultured in 6cm dishes and treatment with POLQ overexpression system or siRNAs. Using a culture medium containing 0.2% FBS, cells were synchronized to the G0 phase by serum deprivation for 16-18 hr, followed by a 24 hr released period. To analyze the cell cycle using FCM, trypsin-treated cells were fixed in 70% ethanol at -20°C for an entire night, rinsed with staining buffer, and stained with propidium iodide (PI) solution (Elabscience, E-CK-A351, China) for 30 min. For further counting the cells were washed with PBS and trypsin-attached. Flow cytometry (BD Biosciences, USA) was used to examine the samples.

**Statistics**

The quantitative data were first tested for normally distribution, the Mann-Whitney U-test or the Wilcoxon rank sum test was utilized to compare the two groups did not satisfy the normal distribution (*p* < 0.05). The Kruskal-Wallis test was utilized to compare the multiple groups did not satisfy the normal distribution (*p* < 0.05), and multiple hypothesis tests were performed by the Bonferroni (Dunn) procedure to correct the level of significance. The t-test was utilized to compare the two groups of variables were close to normal distribution (*p* > 0.05), and the One-way ANOVA test was utilized to compare multiple groups.

**References**

1 Barrett T, Suzek T O, Troup D B, et al. NCBI GEO: mining millions of expression profiles--database and tools. *Nucleic Acids Res*, 2005,33(Database issue):D562-D566.

2 Colaprico A, Silva T C, Olsen C, et al. TCGAbiolinks: an R/Bioconductor package for integrative analysis of TCGA data. *Nucleic Acids Res*, 2016,44(8):e71.

3 Hutter C, Zenklusen J C. The Cancer Genome Atlas: Creating Lasting Value beyond Its Data. *Cell*, 2018,173(2):283-285.

4 Subramanian A, Tamayo P, Mootha V K, et al. Gene set enrichment analysis: a knowledge-based approach for interpreting genome-wide expression profiles. *Proc Natl Acad Sci U S A*, 2005,102(43):15545-15550.

5 Mootha V K, Lindgren C M, Eriksson K F, et al. PGC-1alpha-responsive genes involved in oxidative phosphorylation are coordinately downregulated in human diabetes. *Nat Genet*, 2003,34(3):267-273.

6 Konermann S, Brigham M D, Trevino A E, et al. Genome-scale transcriptional activation by an engineered CRISPR-Cas9 complex. *Nature*, 2015,517(7536):583-588.


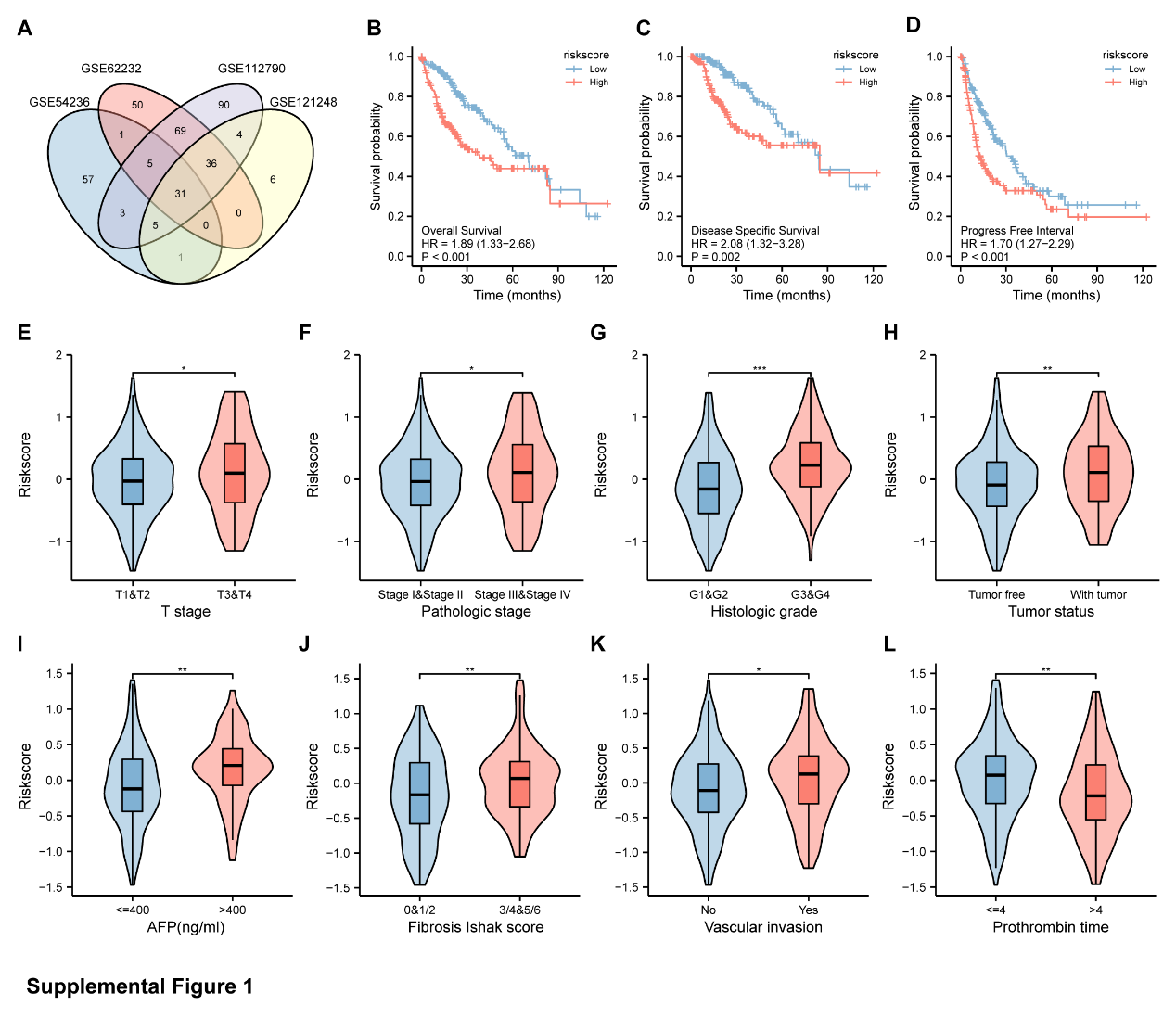


**Supplemental Figure** **1.** Comprehensive analysis for HCC patients with the risk scores. **(A)** Venn diagram of DEGs overlapping datasets GSE54236, GSE62232, GSE112790, and GSE121248. **(B-D)** Kaplan-Meier of OS, DSS, and PFI curve with log-rank test for risk score. **(E-L)** Violin plot showing correlation between risk scores and T-stage, pathological stage, pathological grading, tumor status, the level of AFP, fibrosis ishak score, vascular invasion, and prothrombin time.


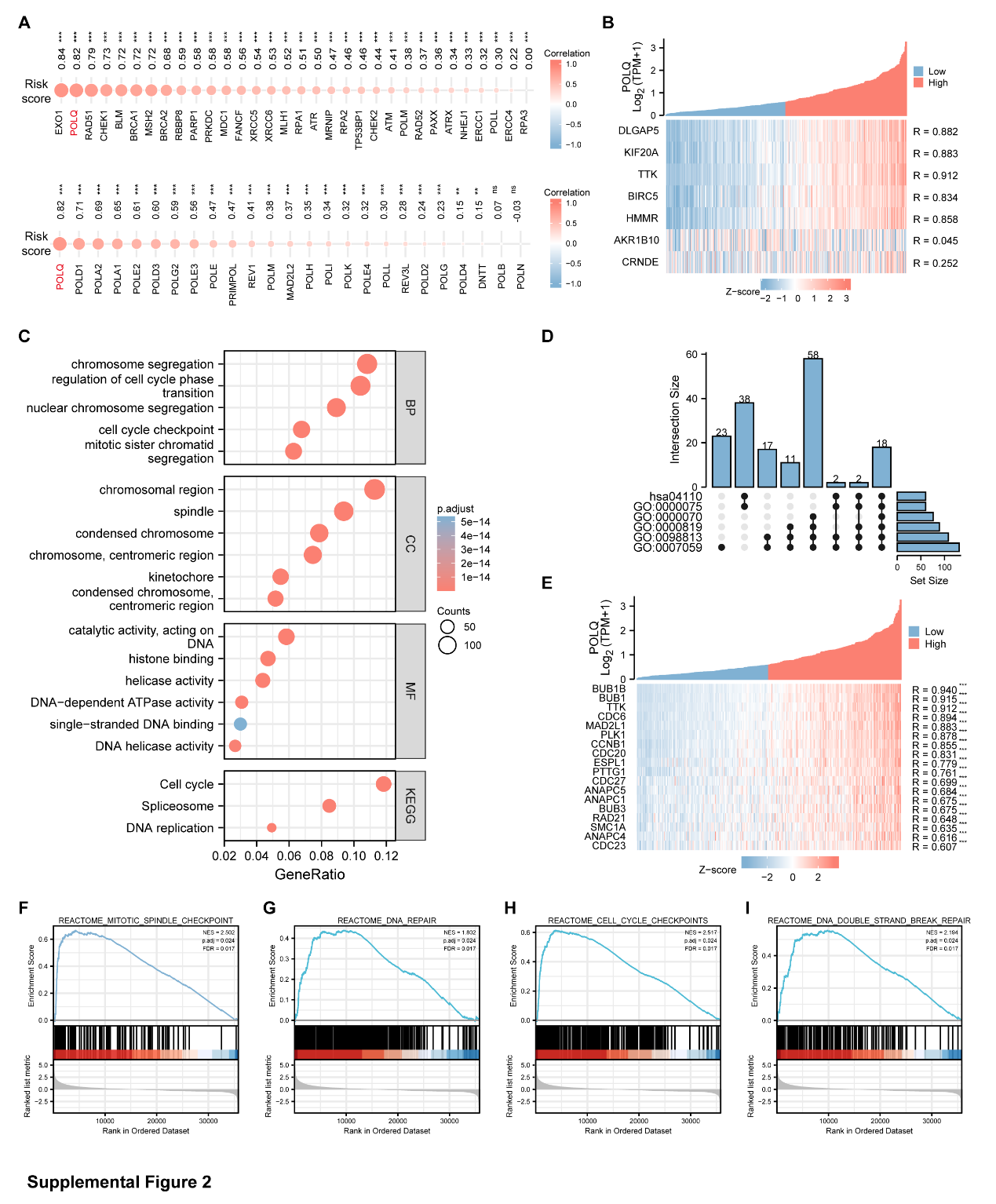


**Supplemental Figure** **2.** Correlation analysis for POLQ and CIN. **(A)** Correlation of bubble plot showing the risk scores of DNA damage repair-related gene and the entire family genes of DNA polymerases. **(B)** Correlations between POLQ and seven key genes of the risk model in TCGA-LIHC cohort. **(C)** GO and KEGG pathway analysis showing the enrichment analysis of POLQ associated genes. **(D)** Upset plot showing the genes associated with relevant terms of functional enrichment and KEGG pathway including chromosome segregation and cell cycle. **(E)** Correlation analysis between POLQ and genes from co-enriched by upset plot. **(F-I)** GSEA analysis of differential genes associated with POLQ, involved aspects including mitotic spindle checkpoint, DNA repair, DNA double strand break repair and cell cycle.


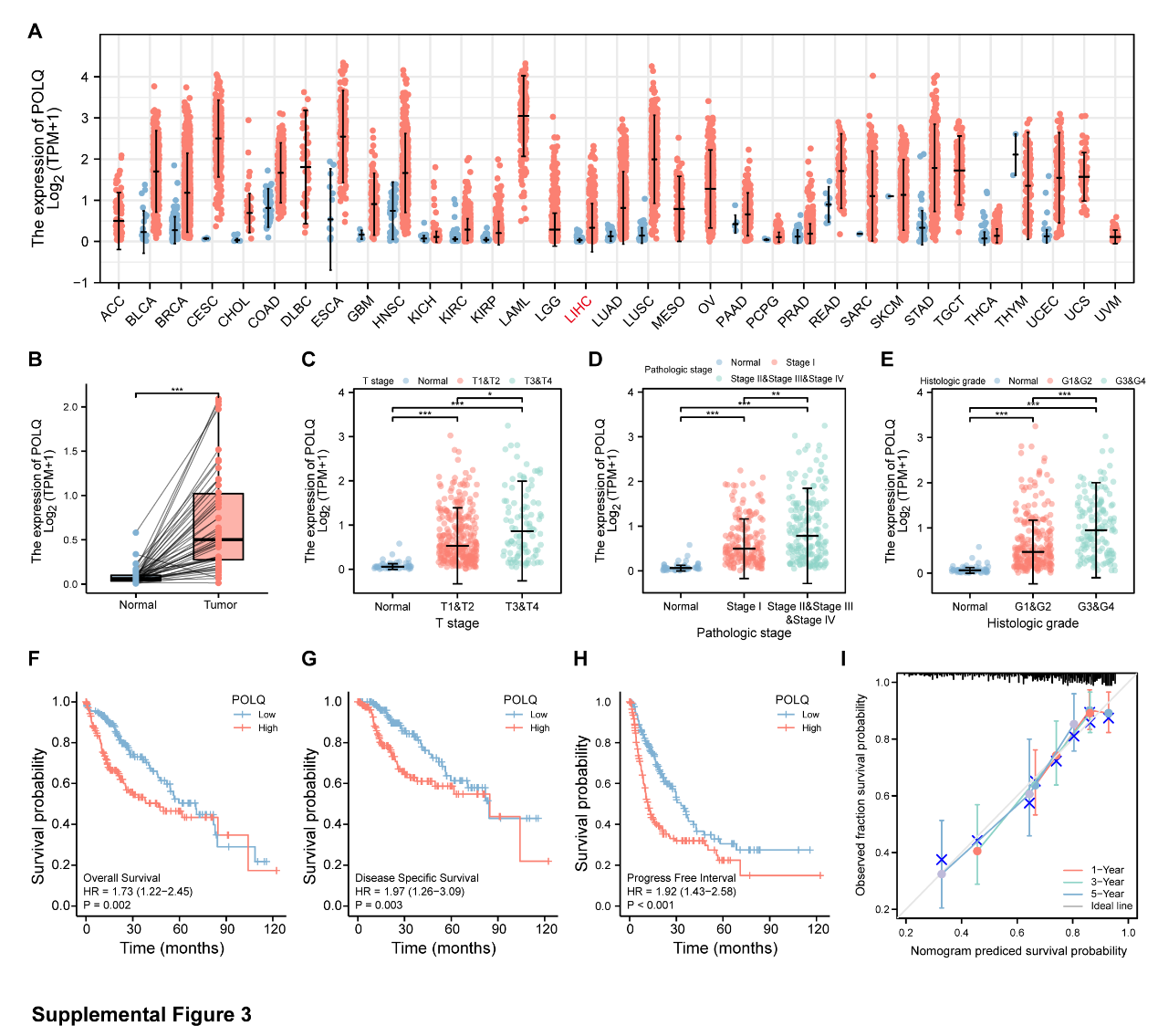


**Supplemental Figure 3.** POLQ was elevated in TCGA-LIHC cohort and associated with poor prognosis of HCC patients. **(A-B)** POLQ expression in different cancer and paired HCC patients. **(C-E)** Expression of POLQ in different T stages, pathological stages and histological grades. **(F-H)** Kaplan-Meier curve analysis of OS, DSS, and PFI based on high and low POLQ expression level. **(I)** The calibration curve for prediction model with a model consistency of more than 0.7. *p* values were shown in the figure, * *p*< 0.05; ** *p*< 0.01; *** *p*< 0.0001.


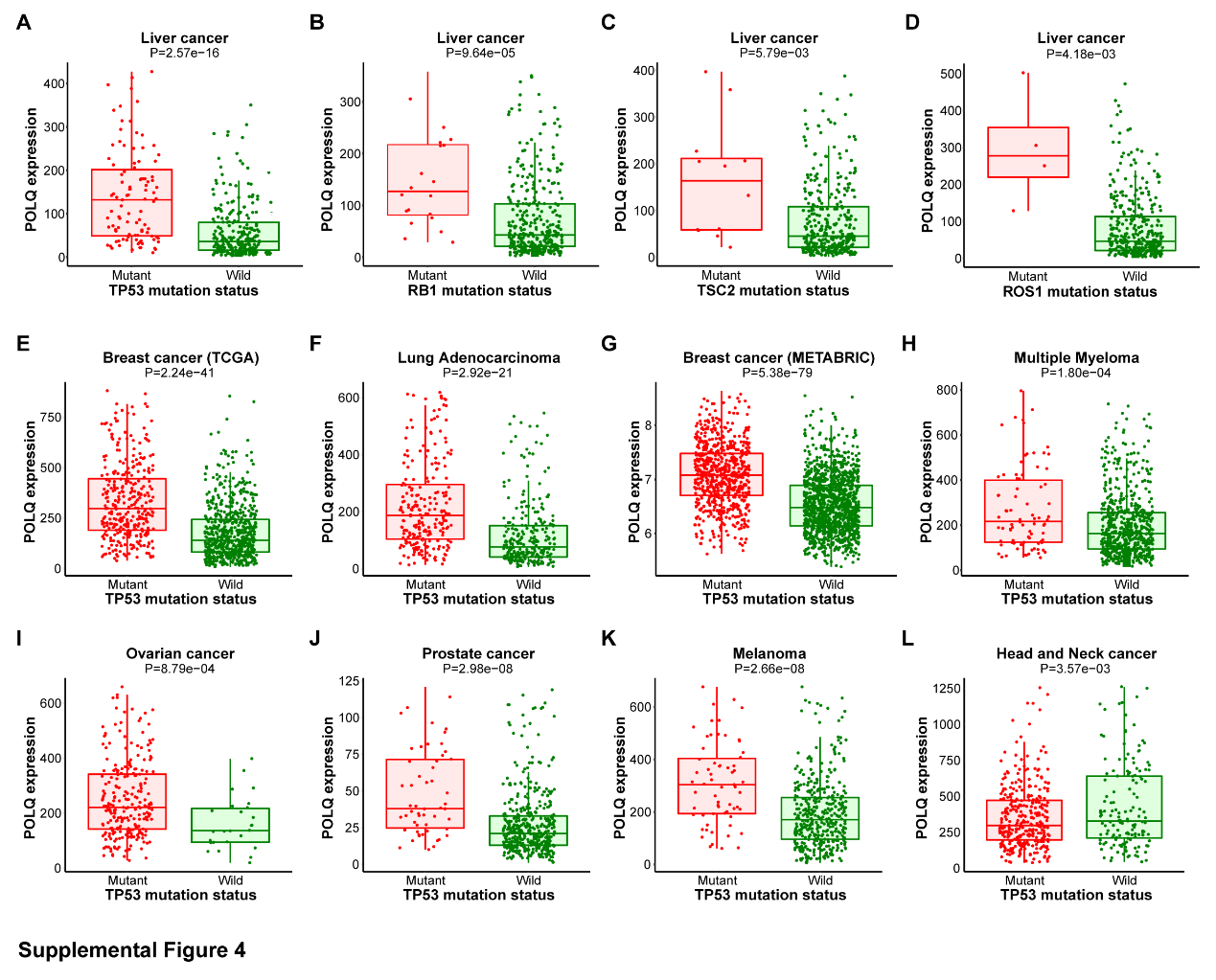


**Supplemental Figure 4.** The association between the expression of POLQ with frequently mutated genes and TP53 mutation status in different cancers. **(A-D)** Effect of key genes (including TP53, RB1, TSC2, and ROS1) mutation on POLQ expression in HCC. **(E-L)** Effect of TP53 mutation on POLQ expression in breast cancer (TCGA), lung adenocarcinoma, breast cancer (METABRIC), multiple myeloma, ovarian cancer, prostate cancer, melanoma, and head and neck cancer.


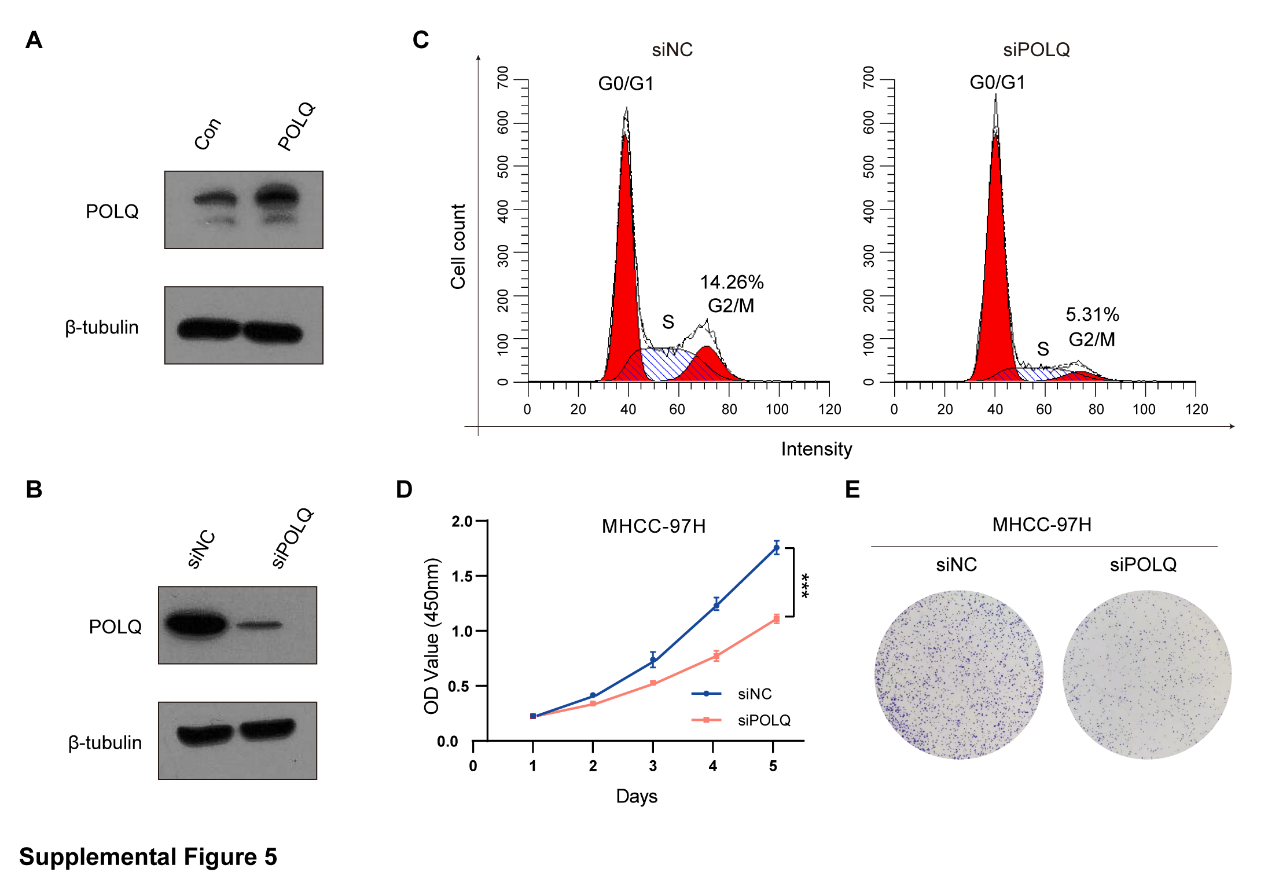


**Supplemental Figure 5.** Effect of overexpression and silencing POLQ in HCC cell lines. **(A-B)** Western blot analysis to detect the expression of POLQ in PLC/PRF/5 and MHCC-97H after overexpression or silencing. **(C)** The distribution of different cell cycle phases was determined by flow cytometry. Cell cycle analysis revealed that the proportion of G2/M phase significantly decreased in silencing POLQ cells relative to control cells. **(D-E)** CCK-8 assay and clone formation in silencing POLQ cells.

**Supplementary Table 1. siRNA sequence used in this study**

| siRNA | Target sequence (5′ to 3′) |
| --- | --- |
| POLQ siRNA (1) | GGACCATGTTGTTAGTTTA |
| POLQ siRNA (2) | GGACTACTATTGATTGGTA |
| POLQ siRNA (3) | CAAGCACTATTACCAAACA |
| NC siRNA | UUCUCCGAACGUGUCACGUTT |

**Supplementary Table 2.** **Antibody for western blotting and immunohistochemistry.**

| **Antibody** | **Company** | **Cat No.** |
| --- | --- | --- |
| POLQ | ABMART | TD13563 |
| BUB1B | ZENBIO | R26485 |
| BUB1 | ZENBIO | R382350 |
| TTK | HUABIO | HA500249 |
| MAD2L1 | ABMART | MG770617 |
| PLK1 | ABMART | MG670393 |
| β-tubulin | CWBIO | CW0098M |
| HRP-labeled Goat Anti-Rabbit IgG(H+L) | Biosharp | BL003A |
| HRP-labeled Goat Anti-mouse IgG(H+L) | ZSGB | ZB-2305 |
